# Management of the Pediatric Patient with Suspected Diagnosis of Obstructive Sleep Apnea Syndrome

**DOI:** 10.3390/children10071225

**Published:** 2023-07-14

**Authors:** Sorina Savin, Luca Mezzofranco, Antonio Gracco, Giovanni Bruno, Alberto De Stefani

**Affiliations:** 1 Department of Neuroscience, Section of Pediatric Dentistry, University of Padua, 35122 Padua, Italy; 2 Department of Pharmacological Sciences, University of Padua, 35122 Padua, Italy

**Keywords:** screening, pediatric OSAS, respiratory polygraphy, rapid palatal expansion, treatment

## Abstract

Aim: The aim of this paper is to describe the multidisciplinary management pathway for pediatric patients with suspected obstructive sleep apnea syndrome (OSAS) conducted by the Pediatric and Orthodontic Department of the Dental Clinic of Padua. Materials and methods: All pediatric subjects undergo a comprehensive medical history, including the completion of the Pediatric Sleep Questionnaire (PSQ), and a physical examination. Patients with suspected OSAS are placed on a waiting list for home respiratory polygraphy testing. The respiratory polygraphy examination is conducted over two consecutive nights and interpreted by a neurologist. Additionally, patients diagnosed with OSAS undergo a case study involving intraoral and extraoral photography, as well as radiographic evaluation. Results: Between September 2021 and May 2023, a total of 134 subjects (including 76 males), with an average age of 9.2 years, were identified as diagnostic suspects. Among these, 38 patients (28.3%) tested positive based on respiratory polygraphic results. Depending on the severity and etiopathogenetic characteristics of the disease, the positive cases were referred to various specialists. Conclusion: In the daily clinical practice of dentistry, particularly in orthodontics and pedodontics, the establishment of a defined management pathway for pediatric patients with OSAS is crucial. The collaboration of a multidisciplinary team with a shared objective of achieving accurate diagnosis and implementing targeted treatment in a timely manner is essential. Regular re-evaluation of patients through clinical and instrumental examinations is recommended.

## 1. Introduction

Obstructive sleep apnea syndrome (OSAS) is a sleep-related breathing disorder (SRBD) characterized by the repetitive occurrence of partial (hypopnea) and/or complete (apnea) obstruction of the upper airways during the night. This condition can affect individuals at various stages of development, with a prevalence ranging from 1.2% to 5.8%. The main distinguishing factor of OSAS is the recurrent blockage of the air passages, leading to interrupted breathing patterns during sleep. It is important to note that the prevalence and occurrence of this disorder can vary within the specified range depending on the specific population being studied and the demographic factors associated with it [1].

Currently, the diagnosis of obstructive sleep apnea syndrome (OSAS) is often delayed or overlooked, which is concerning because early and accurate diagnosis plays a crucial role in preventing significant systemic complications. This is particularly important for individuals in different stages of development, as the absence of timely diagnosis can result in a wide range of complications, including cardiovascular and metabolic issues, neurocognitive and behavioral disorders, and impaired growth. However, the lack of awareness and effective interdisciplinary collaboration regarding the diagnosis and treatment of OSAS remains a significant challenge. It is, therefore, of utmost importance to focus on educating and raising awareness among various healthcare professionals. This will enable prompt intervention and enhance the quality of life for affected individuals. By increasing awareness and fostering collaboration, we can ensure the timely diagnosis and appropriate management of OSAS in pediatric patients, thereby mitigating potential long-term consequences and improving overall outcomes [2].

According to the American Academy of Pediatrics (AAP), it is recommended to include a comprehensive assessment of sleep habits, family history, and clinical examination as part of routine medical visits. This approach aims to facilitate an initial screening for potential patients with obstructive sleep apnea syndrome (OSAS). By incorporating a thorough evaluation of sleep-related issues, gathering relevant family medical history, and conducting a comprehensive clinical examination, healthcare providers can identify individuals who may be at risk for OSAS. This initial screening process helps in determining whether further diagnostic testing or referral to a sleep specialist is necessary for a definitive diagnosis and appropriate management of the condition. By implementing these guidelines, healthcare professionals can improve the identification and timely intervention for patients with OSAS, thereby reducing the potential impact of the condition on their overall health and well-being [1].

In addition to the general medical history, validated questionnaires should be administered to parents [3,4,5], including the PSQ (Pediatric Sleep Questionnaire) [6]. This questionnaire was developed for children and adolescents between 2 and 18 years old, and its Italian version was validated by Cozza et al. [7]. The PSQ turns out to be the most accurate questionnaire among those in the literature [8] and is a valid diagnostic aid in identifying subjects with a high risk of sleep breathing disorders [9].

The presence of risk factors can raise suspicion for obstructive sleep apnea syndrome (OSAS); however, a definitive diagnosis typically requires the use of instrumental tests, such as polysomnography. Polysomnography is considered the gold standard for diagnosing OSAS. It involves monitoring various physiological parameters during sleep, including brain activity, eye movements, muscle tone, heart rate, oxygen levels, and respiratory effort. The data collected from polysomnography provide valuable information about the severity and characteristics of sleep-related breathing disturbances, which helps in determining the appropriate treatment plan. Additionally, other diagnostic tools, such as home sleep apnea testing and portable monitoring devices, may be utilized in specific cases to assess OSAS. These diagnostic tests, in combination with a thorough clinical evaluation, aid in achieving an accurate diagnosis and guiding the management of OSAS. This comprehensive diagnostic test provides detailed information on sleep patterns, the presence of apnea and hypopnea events, and various other related variables. Moreover, in certain cases, home respiratory polygraphy, which is a simplified version of polysomnography, can also be used for diagnosis. While it involves a reduced set of monitoring parameters, it still offers valuable insights into breathing patterns and sleep disruptions. These instrumental tests play a crucial role in confirming the presence and determining the severity of OSAS. They enable healthcare professionals to develop tailored and appropriate treatment plans for patients, taking into account the specific characteristics of their condition. By utilizing these diagnostic tools, healthcare providers can effectively manage and improve the quality of life for individuals affected by OSAS [10].

Currently, respiratory polygraphy, also known as cardiorespiratory monitoring, is becoming increasingly preferred for the diagnosis of OSAS. This is due to several reasons, including comparable results to polysomnography and several advantages in terms of timing, cost, and patient accessibility. Respiratory polygraphy offers comparable diagnostic accuracy to polysomnography while being less time-consuming and more cost-effective. It involves the monitoring of essential parameters such as respiratory effort, airflow, oxygen saturation, and heart rate. By focusing on these key variables, respiratory polygraphy can effectively detect and evaluate sleep-related breathing abnormalities associated with OSAS. Moreover, respiratory polygraphy devices are often portable and user-friendly, allowing patients to undergo testing in the comfort of their own homes. This increased accessibility eliminates the need for overnight stays in sleep laboratories, making it a more convenient option for patients. While polysomnography remains the gold standard for comprehensive sleep assessment, respiratory polygraphy has emerged as a valuable alternative for the initial screening and diagnosis of OSAS. It offers a practical and efficient approach that maintains diagnostic accuracy while addressing practical considerations such as time, cost, and patient comfort. However, the final decision on which diagnostic method to use should be made by healthcare professionals based on individual patient characteristics and clinical considerations [11,12].

Respiratory polygraphy enables the recording of a limited set of channels, including nasal flow via a nasal cannula, breathing noise through a microphone, body position using an accelerometer, oxygenation and heart rate via a pulse oximeter, and thoracic and abdominal movements through elastic bands. In contrast, polysomnography encompasses the aforementioned parameters recorded during respiratory polygraphy, while also including additional measurements such as an electroencephalogram (EEG), an electromyogram (EMG), and an electrooculogram (EOG). These additional measurements play a vital role in determining the child’s sleep status and identifying specific sleep stages. The EEG records brain activity, enabling the identification of sleep stages such as rapid eye movement (REM) and non-rapid eye movement (NREM) sleep. The EMG measures muscle activity, including the chin and limb muscles, aiding in the detection of changes in muscle tone during sleep. The EOG tracks eye movements, providing insight into eye activity and assisting in identifying sleep stages. By incorporating these supplementary channels, polysomnography offers a more comprehensive assessment of sleep architecture and facilitates the identification of specific sleep disorders or irregularities. Nonetheless, polysomnography is more intricate and resource-intensive compared to respiratory polygraphy, making it suitable for comprehensive sleep evaluations and in-depth investigations. The dentist is, therefore, in an excellent position to recognize any signs and symptoms of sleep apnea [13], as he has the opportunity to visit children regularly, through check-ups, from the tender age of two. The objective of this article is to describe the path of pedodontists and orthodontists in the management of patients with suspected OSAS, starting from the diagnosis up to the multidisciplinary therapeutic choice [14].

## 2. Materials and Methods

The study protocol was approved by the Ethical Committee of the University of Padua (protocol numb.3493/AO/15). All pediatric and adolescent patients at the Dental Clinic of Padua, Department of Pedodontics and Orthodontics, undergo a comprehensive general anamnesis, which includes detailed inquiries about the quality of sleep. The primary objective is to identify potential risk factors associated with obstructive sleep apnea syndrome (OSAS), such as allergies or congenital syndromes like Down syndrome, Prader–Willi syndrome, and others. In addition to the general anamnesis, patients are also requested to complete the Pediatric Sleep Questionnaire (PSQ). This questionnaire comprises a series of 22 questions, each offering three possible answer options: “yes”, “no”, and “I don’t know”. The PSQ is designed to assess various aspects related to sleep and breathing patterns. To calculate the PSQ score, the number of questions answered positively (i.e., “yes”) is divided by the sum of questions answered both positively and negatively (i.e., “yes” + “no”), excluding questions with an uncertain answer (i.e., “don’t know”). The resulting score falls within the range from 0 to 1, with a score greater than 0.33 indicating a high risk of sleep-related breathing disorders. By incorporating the comprehensive general anamnesis and the utilization of the PSQ, the Dental Clinic of Padua ensures a thorough evaluation of pediatric and adolescent patients, enabling the identification of potential risk factors and the early detection of sleep-related breathing disorders, including OSAS. To ensure a comprehensive evaluation, a physical examination of the young patient is also performed. This examination helps assess various clinical factors that may contribute to or indicate the presence of sleep-breathing disorders. By combining the information obtained from the anamnesis, PSQ, and physical examination, the dental clinic can gather a thorough assessment of the patient’s sleep health. This approach allows the dental team to identify potential cases of OSAS early on, enabling timely referral for further evaluation and management. By integrating the assessment of sleep-related factors into routine dental care, the Dental Clinic of Padua aims to contribute to the overall well-being of their pediatric and adolescent patients. 

**Figure 1:**
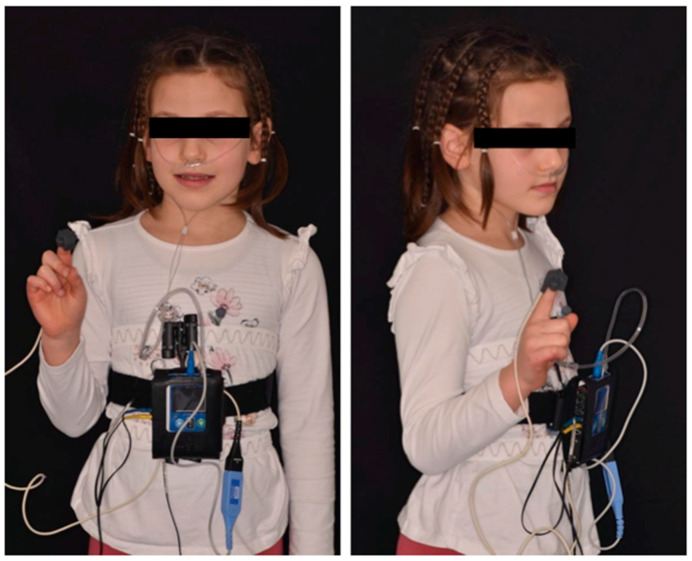
Embletta respiratory polygraphy: nocturnal cardiorespiratory monitoring.

Children with risk factors for OSAS are placed on a waiting list for the execution of home respiratory polygraphy to confirm or not the diagnosis of OSAS. The SapioLife Embletta respiratory polygraphy (Figure 1) is used for the diagnostic assessment, and its diagnostic accuracy has been validated through comparisons with polysomnography (PSG) [15]. To properly program the device, the patient’s anthropometric measurements, as well as the scheduled sleep and wake times for the examination days, are required. Parents of the patients are provided with detailed instructions on how to use the device, and they are given the opportunity to practice positioning it on their child while in the dental clinic. This practical demonstration aims to address any doubts or concerns they may have regarding the device’s usage. Additionally, parents are requested to fill out an evening diary and a morning diary. These diaries are useful for the neurologist in identifying any incorrect sleep hygiene behaviors that may be affecting the child’s sleep. The examination is conducted over two consecutive nights to enhance the chances of obtaining a successful recording with reliable data for analysis and interpretation. Ultimately, the recording with the longest trace and fewer artifacts is selected for further analysis. The neurologist evaluates several parameters, including the apnea–hypopnea index (AHI) per hour of sleep, AHI index in the supine position, number of desaturation events per hour of sleep (ODI), average oxygen saturation (average SaO_2_), minimum recorded saturation (NADIR), and cumulative percentage of time spent at SaO_2_ < 90% (CT90). Based on the values of these parameters, patients are classified according to the guidelines established by the Italian Association of Sleep Medicine (AIMS) as non-pathological, minimal OSAS, mild OSAS, moderate OSAS, or severe OSAS. This classification system aids in guiding treatment decisions and management strategies. By utilizing these parameters and following the guidelines, the dental clinic aims to accurately diagnose and classify patients with OSAS, thereby enabling appropriate interventions and personalized care plans for each individual (Table 1).

Subjects who test positive on the respiratory polygraphic assessment undergo the collection of photographic documentation to conduct a more detailed study of their extraoral and intraoral characteristics. This study aims to delve into specific morphological aspects that may predispose individuals to OSAS. These aspects include occlusal and craniofacial features, adenotonsillar hypertrophy, ankyloglossia (tongue-tie), atypical swallowing patterns, and more [14].

Furthermore, to complete the comprehensive assessment of the case, specific radiographs are required. An orthopantomogram (OPG) is taken to evaluate the dental status and examine adjacent structures. A lateral cephalometric teleradiograph is performed to conduct a cephalometric analysis and study the various positions of upper airway obstruction. In certain cases, a cone beam computed tomography (CBCT) scan may also be necessary to provide a detailed study of the upper airways. These radiographic investigations, along with photographic documentation, contribute to a thorough understanding of the patient’s anatomical and structural characteristics that are relevant to OSAS. These additional diagnostic tools help healthcare professionals evaluate the patient’s dental and craniofacial features, as well as the upper airway anatomy. By assessing these aspects, the dental clinic can gain valuable insights into the underlying factors contributing to OSAS and develop appropriate treatment plans tailored to each patient’s specific needs.

They help identify any underlying factors that may contribute to airway obstruction and assist in the formulation of an appropriate treatment plan. By utilizing a multidimensional approach that incorporates respiratory polygraphy, photographic documentation, and radiographic studies, healthcare professionals can gain valuable insights into the patient’s condition. This comprehensive evaluation enables a more accurate diagnosis and facilitates the development of individualized treatment strategies for patients with OSAS.

## 3. Results

**Figure 2:**
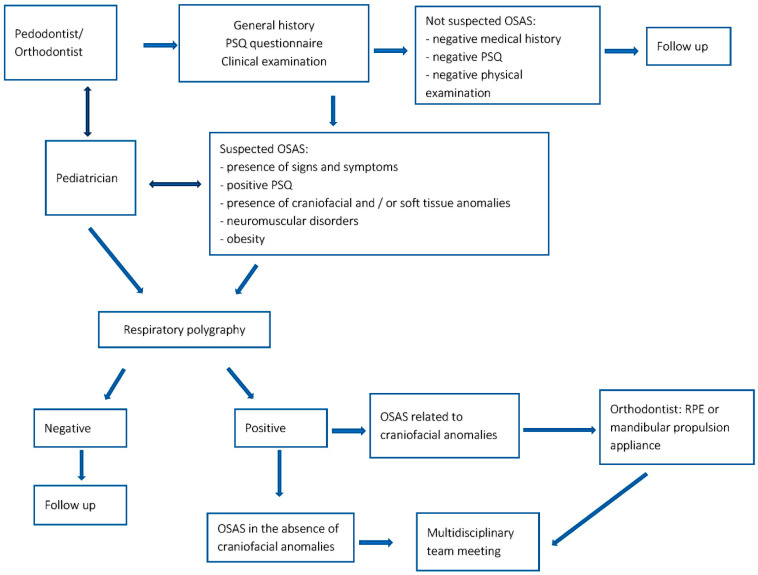
Flowchart on the screening and management pathway of 134 children with suspected OSAS intercepted by orthodontists and pedodontists.

**Figure 3:**
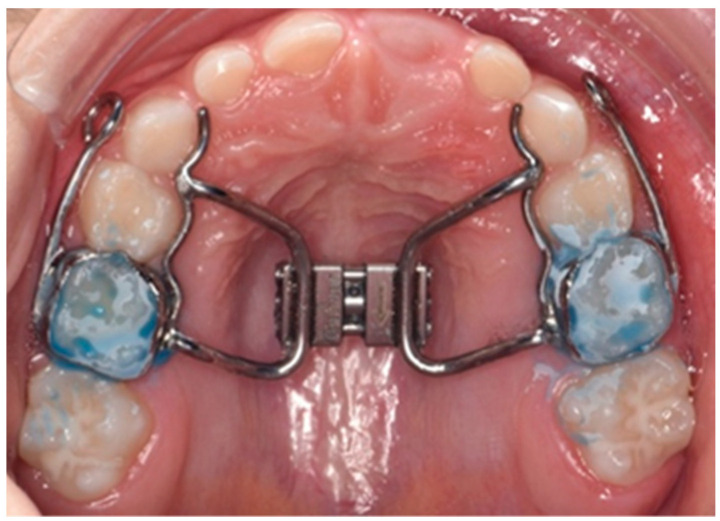
Rapid palatal expander with bands on deciduous molars and arms for Delaire.

By following the flowchart presented in Figure 2, a total of 134 children (76 males) with an average age of 9.2 years were identified as diagnostic suspects at the Dental Clinic of Padua’s pedodontic and orthodontic department. This observation period spanned from September 2021 to May 2023. All 134 patients with suspected diagnosis underwent respiratory polygraphy testing, and 38 of 134 (28.3%) tested positive for OSAS. Children with OSAS had different degrees of severity and different pathogenic factors, according to which they were referred to different specialists. A total of 13 out of 38 had maxillary growth alteration, dental crowding, unilateral or bilateral crossbite, and Class III malocclusion. These patients were treated immediately by the orthodontist with the Rapid Palatal Expander (RPE) (shown in Figure 3). Respiratory polygraphy was routinely performed after orthodontic treatment. The treatment approach consisted of two steps: firstly, orthopedic expansion of the upper jaw was performed to address the malocclusion. Secondly, respiratory polygraphy was conducted to assess improvements in respiratory problems. The results indicated positive changes, including improvements in the apnea-hypopnea index (AHI) values and an increase in average oxygen saturation.

Out of the thirteen children, six experienced a complete resolution of the syndrome, as indicated by an AHI value of less than 1. It is important to note that these children were still awaiting other treatments, such as adenotonsillectomy or the results of gradual weight loss. In the remaining subjects who underwent orthopedic expansion of the upper jaw, optimal resolution of the syndrome was not achieved. However, the orthodontic treatments still resulted in a significant increase in peripheral oxygenation. These findings suggest that orthodontic interventions, specifically the orthopedic expansion of the upper jaw, can have positive effects on respiratory problems associated with sleep-related breathing disorders. While complete resolution may not be achieved in all cases, improvements in AHI values and oxygen saturation can still be observed. As a result, the orthodontist, who is qualified and certified in the prevention, diagnosis, and treatment of OSAS collaborates with other specialists, including ENT (Ear, Nose, and Throat) specialists, nutritionists, and speech therapists, based on the severity of the syndrome. Following orthodontic treatment, proper coordination of muscle forces is crucial for maintaining treatment results and promoting nasal breathing. Within our team, the Postgraduate School of Pediatric Dentistry has developed a book specifically designed for children, which provides instructions on myofunctional exercises that can be performed at home. The book includes written explanations as well as a QR code that links to illustrative videos demonstrating the exercises. These exercises focus on nasal breathing, atypical swallowing, and other relevant aspects, strengthening the connection between the orthodontist’s treatment and the speech therapist’s intervention. By incorporating these myofunctional exercises into the treatment plan, the orthodontist and speech therapist work together to improve breathing patterns, swallowing functions, and overall oral health. This interdisciplinary approach ensures a comprehensive and integrated approach to the management of OSAS. This multidisciplinary approach ensures that patients receive appropriate and specialized care from the respective healthcare professionals involved in the management of OSAS. By identifying and referring these patients to the appropriate specialists, the Dental Clinic of Padua aims to ensure comprehensive and targeted treatment strategies for individuals affected by obstructive sleep apnea. This collaborative approach enhances the quality of care provided to these patients, promoting a better management of their condition and improving their overall well-being.

## 4. Discussion

Snoring, sleep apnea, oral breathing, and dry mouth upon awakening are the most commonly reported factors by parents of individuals with OSAS [16]. Therefore, incorporating specific sleep-related questions and validated questionnaires helps identify potential diagnostic suspects and raises awareness among parents regarding this issue.

Regarding the extraoral facial evaluation, subjects with OSAS often exhibit characteristics of adenoid facies, which are typical of oral respiratory patients. These characteristics include an increased lower third of the face, dark circles, flattened cheekbones, dry lips, labial incompetence, a wide mandibular angle, underdeveloped nasal bones, and pronounced nasolabial furrows. During the intraoral evaluation, the presence of a contracted palate with an ogival and deep shape is frequently observed, along with posterior and/or anterior crossbite, adenotonsillar hypertrophy, mandibular hypoplasia, Class II malocclusion, wear on deciduous and/or permanent dentition, dental crowding, or reduced Leeway Space. By recognizing these features through comprehensive evaluations, healthcare professionals can contribute to the early identification and management of OSAS in individuals. This holistic approach helps address the specific oral and facial characteristics associated with the condition, promoting better overall health and well-being for patients [13,17].

Once the severity of OSAS has been determined and phenotypic and occlusion characteristics have been studied, appropriate treatment can be directed for the child, involving different specialists (as shown in Table 2) based on the individual case.

The therapeutic approach for pediatric patients with OSAS is typically gradual. For subjects with obesity, the initial step is a referral to a dietician for weight loss, often in combination with other therapies. Patients with nasal obstruction are referred to an otolaryngologist, and proper nasal hygiene instruction and the use of nasal steroids are often recommended. In cases of moderate-to-severe OSAS with a predominant obstructive component, the primary therapeutic option is adenotonsillectomy. In such cases, all photographic documentation and the respiratory polygraphic report are sent to the otolaryngologist. Evaluation by the otolaryngologist should always be accompanied by fibroscopy of the upper airways. Simultaneously, if occlusal problems are present, early orthodontic intervention is attempted to address the associated malocclusion and reduce the severity of the condition while awaiting ENT surgery. Patients with mild-to-moderate OSAS and occlusal or craniofacial problems undergo the most appropriate orthodontic treatment to improve the malocclusion and, subsequently, the respiratory problem. Several studies support the effectiveness of treatment with RPE in increasing nasal volume and respiratory airflow. By involving the relevant specialists and implementing a comprehensive treatment approach, healthcare professionals aim to address both the occlusal and respiratory aspects of OSAS in pediatric patients. This multidisciplinary approach ensures that appropriate interventions are provided to improve the malocclusion, nasal obstruction, and overall respiratory function, promoting a better quality of life for the affected individuals [18,19,20]. The combination of RPE and Delaire facemask does not have substantial evidence supporting its effectiveness. However, several studies in the literature have demonstrated a reduction in the apnea–hypopnea index (AHI) with the use of mandibular advancement devices (MADs) for mandibular retrusion in subjects with OSAS [21]. Additionally, for correcting Class II malocclusions, the use of mandibular thrusters has been shown to contribute to a reduction in the AHI index. In cases wherein myofunctional therapy is required, the involvement of a speech therapist is beneficial. Literature reports indicate that myofunctional therapy can lead to a significant reduction in the AHI index, with a 50% reduction observed in adults and a 62% reduction in children. While further research is needed to evaluate the efficacy and combination of certain treatment modalities for OSAS, current studies suggest that mandibular advancement devices and myofunctional therapy can be valuable in reducing the severity of the condition and improving overall respiratory function. Collaborative efforts between healthcare professionals, including orthodontists, speech therapists, and specialists in sleep medicine, can provide comprehensive care and maximize treatment outcomes for patients with OSAS [22]. Myofunctional treatment can be combined with RPE treatment to maintain the positive outcomes of orthodontic intervention and restore normal nasal breathing. This combination approach has shown promising results in the literature [23,24]. Additionally, if other treatments are ineffective, the use of continuous positive airway pressure (CPAP) therapy should be considered. For patients with central respiratory problems, referral to a neurologist is necessary, providing all the collected documentation and, if possible, a video recording of the child during sleep. Following any type of treatment, follow-up respiratory polygraphy is always conducted to assess the resolution of the pathology. Patients also undergo careful long-term monitoring through clinical and instrumental examinations to ensure optimal outcomes and ongoing management of their condition. Regular evaluations are essential to track the progress and overall effectiveness of the chosen treatment approach.

## 5. Conclusions

Orthodontists and pedodontists play a crucial role in identifying children with OSAS and implementing appropriate treatment interventions to improve or resolve their condition. The diagnostic role of orthodontists and pedodontists is depicted at the beginning of the flowchart in Figure 2. The diagnostic process involves the administration of validated questionnaires (such as the PSQ) to parents, gathering general history information, and conducting a clinical examination. These steps help identify potential risk factors associated with obstructive sleep apnea syndrome (OSAS), including positive responses on the PSQ questionnaire, allergies, congenital syndromes, hyperactivity, lack of concentration, snoring, sleep apnea, and diuresis. An extraoral examination can reveal adenoid facies, which is typical in individuals with OSAS. Additionally, an intraoral examination conducted by the orthodontist and pedodontist is crucial for identifying specific indicators such as a contracted palate with an ogival and deep shape, posterior crossbite, anterior crossbite, tonsillar hypertrophy, dental and/or skeletal malocclusion, and dental crowding. The diagnostic pathway concludes with the confirmation of diagnosis through respiratory polygraphy. By following this flowchart, 28.3% of subjects in a group of 134 patients identified as diagnostic suspects were ultimately diagnosed with OSAS. By employing this systematic approach, orthodontists and pedodontists play a vital role in the early identification and diagnosis of OSAS. Through the integration of questionnaires, clinical examinations, and respiratory polygraphy, they contribute to the accurate diagnosis and subsequent management of the condition. By promptly recognizing the signs and symptoms of OSAS, the risk of complications arising from untreated or mismanaged cases can be minimized. 

The flowchart continues to show the importance of creating a multidisciplinary treatment team. The orthodontist, qualified and certified in the prevention, diagnosis, and treatment of OSAS, meets and coordinates with other specialists. Orthodontists promptly treat the malocclusion with rapid maxillary expansion and mandibular advancement. Firstly, orthodontic treatment resolves the malocclusion. Secondarily, respiratory polygraphic results suggest improvements in respiratory problems: AHI values improvements and average oxygen saturation increase. In the case of OSAS-related craniofacial and occlusal morphologies associated with a history of snoring, mouth breathing, obesity, or allergies, the pediatrician should be involved, and the collaboration of the ENT specialist, speech therapists, nutritionist, and other specialists is required based on the severity of the syndrome and etiopathogenesis. The orthodontist and the pedodontist need this multidisciplinary team, with specialists that must undergo increasingly specialized training and continuing education for the medical profession. This interdisciplinary approach ensures that all aspects of the patient’s condition are addressed, leading to optimal outcomes. By following a well-defined pathway, healthcare providers can streamline the diagnostic process, expedite the confirmation of OSAS, and implement appropriate treatments promptly. 

Early intervention and a coordinated team effort contribute to the successful management of OSAS in children, improving their overall well-being and reducing the risk of long-term complications.

## Figures and Tables

**Table 1:** Classification of OSAS severity (AIMS Guidelines).

Minimal OSAS	AHI 1–3 Events/h and SaO_2_ > 97%
Mild OSAS	AHI 3–4 events/h and SaO_2_ > 97%
Moderate OSAS	AHI 5–9 events/h and SaO_2_ > 97%
Severe OSAS	AHI ≥ 10 events/h and SaO_2_ < 95%

**Table 2:** Professional figures involved in the management of pediatric OSAS treatment.

Problem	Specialist	Treatment
Obesity	**Dietician** **Endocrinologist**	Weight management
Nasal obstruction	**Otolaryngologist**	- Medical therapy - Adenotonsillectomy
Dental and/or skeletal malocclusions	**Orthodontist** **Maxillofacial team**	- Rapid palatal expansion - Mandibular thrusters
Orofacial muscular dysfunctions	**Speech therapist**	Orofacial muscle re-education
- Severe OSAS with no indication for surgical treatment - Residual OSAS after surgery - Delayed surgical treatment	**Pulmonologist**	Positive pressure ventilation with CPAP

## Data Availability

Not applicable.
